# Faster fowl fall frequently: speed and force regulation during turning maneuvers by guinea fowl on high and low friction terrains

**DOI:** 10.1242/jeb.250929

**Published:** 2026-03-30

**Authors:** Hannah Goldsmith, Jade Hall, Monica A. Daley

**Affiliations:** ^1^ Royal Veterinary College, Hatfield AL9 7TA, UK; ^2^ Department of Ecology and Evolutionary Biology, Dunlop School of Biological Sciences, University of California, Irvine, CA 92697, USA; ^3^ Department of Biomedical Engineering, Henry Samueli School of Engineering, University of California, Irvine, CA 92697, USA; ^4^ Department of Mechanical and Aerospace Engineering, Henry Samueli School of Engineering, University of California, Irvine, CA, 92697, USA

**Keywords:** Biomechanics, Bipedal, Maneuvering, Stability, Unsteady locomotion

## Abstract

Safely navigating variable terrains requires animals to balance competing demands of speed, stability, maneuverability and injury avoidance. Straight-line locomotion has been extensively studied, but less is known about how animals coordinate turning maneuvers. The physics of turning creates a coupling between speed, turn sharpness and ground reaction force (GRF) demands, resulting in a trade-off between speed and maneuverability. Here, we investigated locomotor strategies as guinea fowl navigated turns in high and low friction substrates. We measured center of mass trajectories and GRF in four conditions: control straight, control turns, slippery straight and slippery turns. We hypothesized that guinea fowl would slow down in turns to maintain peak GRF similar to that in steady, straight conditions, and that slippery terrain would lead to a shift towards slower speeds and shallower turn angles for slip avoidance. We found that guinea fowl slowed down by 14% in high friction turns and 27% in slippery turns compared with straight running and maintained GRF peaks within the 95% prediction interval for straight runs. Contrary to predictions, guinea fowl used similar turn strategies in low and high friction terrain, executing gradual turns with ∼7 deg change in heading per step, shifting from aerial to grounded running and leaning into the turn. Substantial individual variation in preferred speeds persisted across terrains, and preferred speed correlated with slip and fall rates (faster birds fell more frequently), suggesting individual variation in risk tolerance. Our findings support the hypothesis that animals modulate speed and ground reaction forces to balance competing mechanical demands in unsteady maneuvers, although the underlying control mechanisms remain to be determined.

## Teaser

**Summary:** Birds run slower and modulate limb–substrate reaction forces when executing turns on slippery terrain, but individual speed preferences persist even with practice and experience of higher slip and fall frequency.

## INTRODUCTION

Navigating variable terrains requires animals to balance competing demands of speed, maneuverability, stability and injury avoidance. While mechanisms for stable straight-line locomotion have been studied across diverse taxa (Ferris et al., 1998, 1999; Daley et al., 2006; Grimmer et al., 2008; Sponberg and Full, 2008; Daley et al., 2009; Müller and Blickhan, 2010; Voloshina et al., 2013; Birn-Jeffery et al., 2014; Voloshina and Ferris, 2015; Druelle et al., 2019), less is known about how legged animals coordinate turning maneuvers. Limb joint function during steady and turning locomotion has been studied in guinea fowl (Kambic et al., 2014; Kambic et al., 2015), but the coordination of whole-body dynamics and ground reaction forces during turns on varied terrains remains poorly understood. Turning maneuvers involve both goal-directed changes in direction and stabilizing adjustments to maintain balance, and typically occur over longer timescales compared with disturbance rejection responses (Jindrich and Full, 1999; Alexander, 2002; Jindrich et al., 2007; Jindrich and Qiao, 2009; Tan and Wilson, 2011; Daley, 2016). Understanding strategies used to execute turns in varied substrate conditions can reveal how biomechanical constraints shape maneuvering behavior.

Animals must regulate limb-substrate forces to achieve unsteady tasks demands. Studies of unsteady straight-line locomotion have revealed functional trade-offs in how forces are regulated to meet demands for speed, economy, stability and safety (Birn-Jeffery and Daley, 2012; Vejdani et al., 2013; Birn-Jeffery et al., 2014; Blum et al., 2014; Daley, 2016). In uneven terrain, guinea fowl actively adjust leg landing posture to avoid large fluctuations in loading, maintaining peak forces within ±0.5 BW of steady values, even at submaximal speeds where forces are well below injury limits (Daley and Usherwood, 2010; Birn-Jeffery and Daley, 2012; Vejdani et al., 2013; Blum et al., 2014).

**Table:** 

**List of symbols and abbreviations**	
Δθ	change the velocity vector heading of the center of mass, measured between the start and end of each step
CS	high friction (control), straight runway
CT	high friction (control) 90 deg turn runway
COM	center of mass of the body
COP	center of pressure of the foot–substrate interaction force
**F**_FA_	ground reaction force in the fore–aft direction, in an anatomical reference frame based on velocity heading
|**F**_H_|	vector magnitude of the horizontal forces=(**F**_FA_^2^+**F**_ML_^2^)^0.5^
**F**_H_:**F**_V_	ratio of horizontal force impulse magnitude to vertical force impulse magnitude during each step
FLA_mid_	frontal plane leg angle in the middle of each step, in an anatomical reference frame
**F**_ML_	ground reaction force in the medio–lateral direction, in an anatomical reference frame
**F**_R,max_	peak magnitude of the ground reaction force vector during each step
**F**_V_	ground reaction force in the vertical direction
*J*_ML_	impulse in the medio–lateral direction
*L*_leg_	leg length measured as standing hip height
*L*_leg,mid_	mid stance leg length, as a fractional value relative to standing hip height (*L*_leg_)
LME	linear mixed effects model
*L*_step_	step length, measured as distance traveled between consecutive foot contacts
*M*_b_	body mass
pos_*y*	distance traveled by the bird along the runway, measured at the start of each step
SLA_i_	initial sagittal plane leg angle at the start of each step, in an anatomical reference frame
SS	low friction (slippery) straight runway
ST	low friction (slippery) 90 deg turn runway
*T*_stance_	stance duration
*T*_step_	step duration, measured as time between consecutive foot contacts
*V*_f_	velocity vector of the body center of mass at the end of each step
|*V*_i_|	speed, the magnitude of the velocity vector of the body center of mass at the start of each step
*V*_FA_	velocity component in the fore–aft direction
*V*_ML_	velocity component in the medio–lateral direction

Neurophysiological studies show widely distributed force feedback in the limb, consistent with ability to sense and regulate global limb forces (Nichols, 2018). These findings support the hypothesis that animals regulate limb forces as a control heuristic to balance multiple demands in unsteady movement. Here, we define ‘force regulation’ as maintaining limb forces within the range of steady values for a given speed, even during unsteady movement. Simulations and experiments suggest that force regulation may serve as a ‘good enough’ control strategy that satisfies multiple task demands including: (1) maintaining sufficient body weight support to avoid falls (Daley and Usherwood, 2010; Birn-Jeffery and Daley, 2012; Vejdani et al., 2013; Blum et al., 2014), (2) minimizing fluctuations in muscle force and work, and associated energy cost (Kram and Taylor, 1990; Gutmann and Bertram, 2017a,b), and (3) maintaining loads within safety limits to avoid injury (Biewener, 1982, 1989; Vejdani et al., 2013; Daley, 2016). Force regulation may be a general control principle for stable, robust and safe locomotion. However, force regulation during turning maneuvers could limit speeds and turn sharpness.

**Fig. 1.**
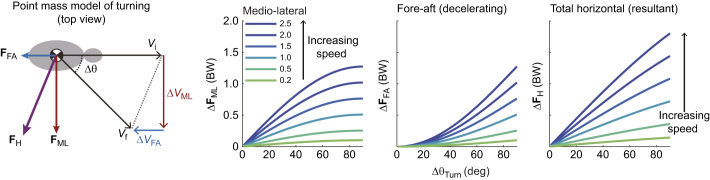
**A point-mass model of turning in the yaw plane, predicting the change in mean ground reaction forces required to achieve a given change in velocity heading per step (Δθ).** Increases in the magnitude of horizontal forces per step were calculated based on impulse–momentum balance (Eqns 1–4), assuming equal speed between initial and final states (*V*_i_=*V*_f_), with lines corresponding to increasing dimensionless speeds. Dimensionless speed is |*V*_i_|/√(g*L_leg_) (McMahon and Cheng, 1990; Daley and Birn-Jeffery, 2018). Mean forces assume a constant stance period of 0.2 s to illustrate representative trends. Turning more sharply requires a larger Δ**F**_ML_, Δ**F**_FA_ and Δ**F**_H_ (graphs illustrated from left to right, respectively). Force demands also increase with speed because of the larger change in momentum for a given change in heading (Daley, 2016). Modified from Daley (2016).

The physics of turning creates a coupling between speed, turn sharpness and ground reaction forces (GRFs). Turning requires increases in medio-lateral forces to change velocity heading, and a simple point-mass model of turning can predict the GRF demands (Fig. 1) (Jindrich and Full, 1999; Alexander, 2002; Jindrich et al., 2007; Jindrich and Qiao, 2009; Tan and Wilson, 2011; Daley, 2016). The model is intentionally abstracted and simplified to illustrate the fundamental mechanical coupling between speed, turn sharpness and force demands based on impulse-momentum balance. Using this framework, horizontal forces in turns relate directly to body mass (*M*_b_), speed (defined as the magnitude of the center of mass (CoM) velocity vector, |*V*|) and the change in velocity heading per step (Δθ) (Fig. 1). If an animal maintains constant speed during a turn, a decelerating force is required along the original heading to avoid a net acceleration (Jindrich and Full, 1999; Jindrich et al., 2007; Jindrich and Qiao, 2009; Daley, 2016). The impulse per step in the medio-lateral (**F**_ML_) and fore-aft (**F**_FA_) directions can be calculated based on impulse–momentum balance from the magnitude of the initial velocity vector (|*V*_i_|) and Δθ, assuming equal initial and final speed (Fig. 1):
(1)

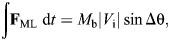

(2)

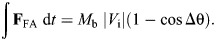


The mean force in each direction can be calculated by dividing impulse by stance duration (*T*_stance_):
(3)

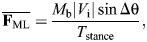

(4)

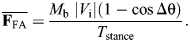


The total increase in horizontal force, |**F**_H_| is the vector magnitude **F**_ML_ and **F**_FA_ (Fig. 1, right). As speed increases, a larger momentum change occurs for a given Δθ, requiring a larger |**F**_H_|. This simple model highlights the tight coupling between speed, turning rate and GRF, and suggests that capacity to exert **F**_H_ against the ground can limit how fast and sharply animals turn (Fig. 1).

Frictional forces at the foot–substrate interface are an additional factor in an animal's ability to exert **F**_H_ to execute turns. (Alexander, 2002; Tan and Wilson, 2011). Frictional force (**F**_f_) between the foot and substrate is equal to the vertical force (**F**_V_) times the coefficient of friction *U*_f_:
(5)

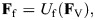


Slips occur when |**F**_H_| exceeds the available frictional force:
(6)

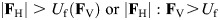


Therefore, the ratio of horizontal to vertical forces, |**F**_H_|: **F**_V_ is the most salient variable for avoiding friction limits – a slip occurs when |**F**_H_|: **F**_V_ exceeds the coefficient of friction between the foot and substrate. Slip risk can be reduced by decreasing **F**_H_, increasing, **F**_V_ or a combination of both. Slip avoidance on low friction terrains may require limiting horizontal forces or adjusting vertical loading to increase available friction.

Turning speed and sharpness can be influenced by multiple mechanical demands in addition to forces. Studies of humans running around circular tracks have found that runners reduce speed while turning in a manner consistent with a leg force limit (McMahon and Greene, 1979; Greene, 1985, 1987; Alexander, 2002; Usherwood and Wilson, 2005). However, in very tight bends, artificially applying an external centripetal force does not allow runners to reach the maximum speeds they achieve in a straight path (Chang and Kram, 2007). This demonstrates that factors beyond leg force limits contribute to reduced speeds in turns. Roll stability can also limit turning, and the maximum centripetal acceleration before reaching the roll limit depends on the ratio of step width to body height (Alexander, 2002). Previous studies on quadrupeds suggest that, compared with bipeds, quadrupeds may have more flexibility to distribute forces among the legs to maintain speed and roll stability while executing turns (Jindrich and Full, 1999; Walter, 2003; Usherwood and Wilson, 2005; Jindrich et al., 2007; Daley, 2016). Collision avoidance can also influence speed in turns – a study of turning quolls found that higher speed in turns was associated with higher risk of collisions, and quolls slowed down with increasing turn sharpness (Wynn et al., 2015). Therefore, a general trade-off between speed and maneuverability exists because of multiple factors, including leg force demands, roll stability limits, and the risk of falls and collisions.

An animal's choice of speed in maneuvers is not only influenced by physical factors, but also by ecological context and perceived risks. The ability to turn quickly is an important factor in maneuvering during predator–prey interactions (Wilson et al., 2013, 2018). Faster is not always better: prey animals may run slower than maximal speeds to enable accurate, safe travel while evading predators. Theoretical models of optimal movement predict a trade-off between speed and safety, whereby faster speed facilitates predator escape, but increases risk of injury (Wheatley et al., 2015). Slips and falls not only increase risk of injury but also increase total travel time owing to delays associated with recovery. Therefore movement speeds can vary depending on the animal's morphology and capacity, environmental risks and the costs associated with mistakes (Wilson et al., 2015; Wynn et al., 2015; Amir Abdul Nasir et al., 2017; Clemente et al., 2019). Experimental measures of animal locomotion have revealed that animals run faster while escaping predators than while foraging, and run faster on wide beams compared with narrow beams, exhibiting context-dependent speed selection (Amir Abdul Nasir et al., 2017; Wheatley et al., 2018; Clemente et al., 2019). However, it remains unknown how rapidly animals adapt movement speed in response to novel terrain conditions that require maneuvers with different levels of risk.

Here, we investigate how guinea fowl adjust speed and limb–substrate forces as they enter turns on high and low friction terrains with differing risk of slips and falls. We compare speeds, COM trajectories and GRFs in four runway conditions: (1) high friction, straight; (2) high friction 90 deg turn; (3) low friction straight; and (4) low friction 90 deg turn. Our first goal is to investigate how birds navigate the trade-off between speed and force in turn maneuvers to test the force regulation hypothesis in the context of submaximal maneuvers. We hypothesize that (H1) guinea fowl will maintain the same relationship between peak |**F**_R_| and speed in turns compared to steady, straight runs. Because peak **F**_V_ increases with speed, increases in **F**_H_ can be offset by slowing down, thereby decreasing **F**_V_ to maintain similar peak resultant magnitude |**F**_R_|. Our second goal is to compare turning on high versus low friction terrains to test how guinea fowl adjust their gait strategy for turns in the presence of increased slip and fall risk. We hypothesize that (H2) slippery terrain will lead to a shift in force regulation to maintain a lower |**F**_H_|:**F**_v_ for slip avoidance. Strategies to achieve this include turning with slower speed and shallower turn angles (Fig. 1). Strategies to minimize slip risk directly conflict with the mechanical demands of turning. Consequently, slippery terrain is expected to limit how fast and sharply guinea fowl turn. We also expect guinea fowl to slow down with practice in low friction turns after experiencing a higher rate of slips and falls.

## MATERIALS AND METHODS

### Animals and training

Seven helmeted guinea fowl [*Numida meleagris* (Linnaeus 1758), four males, three females] were used in this study, which were hatched and reared at the Royal Veterinary College (RVC). Sample size is based on effect size estimates from previous studies on ostriches turning and guinea fowl on slippery terrain (Jindrich et al., 2007; Clark and Higham, 2011). Eight birds were initially trained, but one animal was excluded from the analysis because it refused to cross slippery terrain and therefore had an incomplete dataset. At the time of testing, the guinea fowl were 4 years old with body mass 1.89±0.21 kg and standing hip height 17.53±2.38 cm (means±s.d). Primary feathers were kept clipped to the length of the secondary feathers to prevent flying. Birds were housed as a group and tagged with colored cable ties on the distal tarsometatarsus for identification. Training and experimental data collection were conducted at the Royal Veterinary College, UK (RVC) and ethical approval was granted by the RVC Clinical Research Ethical Review Board (CRERB, protocol URN: 2017 1759-3 to M.A.D.).

### Terrain conditions and runway construction

We recorded trials on four runway conditions: straight and 90 deg turns, each with high and low friction surfaces. These four conditions were designated as control straight (CS), slippery straight (SS), control turns (CT) and slippery turns (ST). Low friction terrain was created using smooth polypropylene sheeting, which was established by Clark and Higham (2011) to successfully elicit a substantial increase in slipping frequency in guinea fowl. The control, higher friction substrate was gritted anti-skid paint specifically designed to increase traction. The runway arena was constructed from plywood, Rexroth metal rods and fittings, and transparent Perspex to allow camera views for high-speed video recordings. The runway was 1 m high, designed to be higher than a guinea fowl's typical maximal jumping height when not aided by flight (∼0.8.m; Henry et al., 2005). The runway was constructed around an array of six 0.9×0.6 m force plates (Kistler type 9287BA, Amherst, NY, United States), oriented with the long side spanning the width of the runway. The straight runway was 1 m wide×9 m long, with a 3.6 m section in the middle containing the six force plates. In the turning runway, a 90 deg turn occurred at the last 0.9 m of the 3.6 m section formed by the sequence of six force plates. The straight section after exiting the corner continued for 2.7 m, forming an ‘L-shape’. In low friction, slippery terrain trials, the entire runway surface was covered with polypropylene sheeting cut to fit the space, with sections over the force plates cut to the exact dimensions of the force platform top plates. At each end of the runway, dark black boxes were adjoined to the runway arena, to allow rest, food and water between trials. The width of the turning runway was wide enough to allow the bird to select a range of paths and turn sharpness as part of its locomotor strategy. This design was intentional, to allow comparison of turning strategy between conditions, to explore how birds prioritize physical demands and performance trade-offs that emerge between speed, turning and terrain friction.

Two AOS high-speed video cameras were positioned facing the runway (S-PRI, Technologies AG, Dättwil, Switzerland), aligned to capture sagittal plane kinematics as the bird ran from one end of the runway to the other. In the turning runway, the cameras were positioned at 90 deg angles, with overlapping views in the corner where turns were executed. Three infrared LED lamps illuminated the runway arena to ensure good video image quality.

### Bird training

Pilot testing trials were performed to acclimate each bird to the laboratory environment and runway locomotion; however, the birds were not exposed to the turning or slippery terrain conditions before data collection. During training, birds were first allowed to rest in a dark box at the end of the runway with food and water. After a few minutes of quiet rest, a vertical sliding door was opened at the front of the box, and the bird encouraged (using waving, clapping and visual motion of looming objects) to run to the opposite end of the runway to another dark box. Once the bird reached the box at the opposite end of the runway, a sliding door was closed, and mealworms provided a positive food reward. The bird was then transferred to the starting box and allowed to rest for several minutes. Through this process, the birds learned to locomote continuously across the runway section to reach the resting boxes at either end.

### Experimental protocol

The birds were exposed to repeated trials of four runway conditions; control straight (CS), slippery straight (SS), control turns (CT) and slippery turns (ST). Trials were collected over a 2 month period in the same sequence for each bird: CT, ST, SS, CS. Consistent sequencing among birds was selected in part for practical reasons because changing the runway configuration was very labor intensive. Considering that the control straight (CS) condition was collected last, there could be some carry over effects from exposure to the manipulated terrain conditions. However, this is expected to lead to conservative estimates for the differences between terrains.

Birds were brought in as pairs each testing day for companionship. While one bird completed trials, the other was placed in a carrier next to the box at the end of the runway to provide additional motivation to reach the resting box. Birds were placed in the starting black box and a small number of practice trials were performed for habituation, until the birds were able to move continuously across the runway from one box to the other. To begin each trial, the cover on the starting box was lifted and the gate was opened. Force and video were synchronously triggered to record for 10s as the bird emerged from the starting box. When the bird reached the end box it was shut, covered, and the bird was transferred back to the starting black box and left to recover for several minutes.

### Inclusion and exclusion criteria

A successful trial was achieved when the bird was able to move continuously across the runway and remain within the runway without contacting the walls, and without excessive wing-flapping or jumping. Each bird varied in both its willingness to participate in continued trials and the consistency of behavior within trials. Trials were repeated until we collected at least ten good continuous locomotion trials, but we stopped data collection if the bird became visibly tired, distressed or uncooperative. More than ten trials were collected when possible, and in these cases, we recorded all trials but sampled a subset of ten trials that were the most continuous and fast trials for that bird, without wall contact events. There were only two cases where we collected the minimum of ten trials, both cases were with different individuals on two different runway conditions. For these cases, we could not institute any selective criteria, and all ten trials were used.

### Digitizing

High-speed video was recorded at 160 frames s^−1^. For each trial, we tracked the sagittal plane kinematics of the approximate body COM along the initial running direction, using DLTdv5 digitizing program (Hedrick, 2008), which runs in MATLAB (MathWorks, Natick, Massachusetts). An initial estimate of the body COM position was obtained by manually tracking a point on the body located approximately 50% along the dorso–ventral axis and 40% along the cranial–caudal axis. This relative position of the guinea fowl body COM was estimated using the suspension technique as described by Fedak et al. (1982). The digitized trials were calibrated and corrected for lens distortion and parallax by digitizing the corners of the six force plates that spanned the runway. The digitized body COM position data were used to estimate the initial condition values (position and velocity) required to double-integrate acceleration from the force plate data to calculate CoM trajectory over time. These initial condition estimates were then refined using the path-match optimization algorithm that minimizes the drift over time of the kinematic versus force-plate derived estimate of COM position over the course of the whole trial (Daley et al., 2006; Blum et al., 2014).

Videos were also manually tracked in ELAN software (version 5.2, 2018: https://archive.mpi.nl/tla/elan; Sloetjes and Wittenburg, 2008) to identify slip, fall and collision events. A slip was defined as an event when the foot moved horizontally along the substrate while flat against the ground during midstance. A fall occurred when another body part, other than the foot, contacted the ground. A collision occurred when the body contacted a wall.

### Data processing to calculate kinetics from ground reaction force data

GRF and center of pressure (COP) data were collected at a sampling frequency of 800 Hz. All data processing was completed using custom scripts in MATLAB. GRF data were corrected for offset and drift by fitting a line to the baseline and subtracting this fitted line from the data. We then applied a fourth-order low pass recursive Butterworth filter with a cutoff frequency of 50 Hz. To reduce the noise inherent in COP data at low forces, the COP trajectories were smoothed using a linear smoothing spline to remove unrealistic transients at the start and end of stance.

Trials were trimmed to correspond to the time-period that the bird was on the force plate array. We then calculated the body COM velocity and position by double integrating the acceleration data, where acceleration was calculated by dividing ground reaction force by body mass (Cavagna, 1975; Daley et al., 2006). Initial conditions for integration were found using the path match optimization technique initially described by Daley et al. (2006). The path match optimization minimizes the sum of the squared error (SSE) between the kinematically estimated paths and the force-plate derived COM paths, to avoid drift due to errors in the initial position and velocity estimates. The optimization function ‘fminsearch’ in MATLAB was used to iteratively adjust the initial condition estimate to minimize the drift between the two trajectory estimates. This method allows within-stride deviations between the kinematic and force-plate derived trajectories. Deviations between these trajectories are expected because kinematic estimates are based on fixed anatomical landmarks, not the true COM. The optimization minimizes the drift between the two trajectories over time, which results from error in initial condition estimates. We used the digitized kinematics for the fore–aft and vertical COM trajectory estimates but used the smoothed COP trajectory to estimate the medio–lateral initial conditions, because the video was only digitized for the sagittal plane. However, initial conditions for the medio–lateral direction are expected to be close to zero because the birds initially run in a straight line. Our final COM trajectory calculations are based on the integrated 3D ground reaction forces, enabling us to measure changes in velocity heading in three dimensions over the course of the trial. We conducted a check on the maximum deviation between the kinematic and kinetic COM trajectories and flagged any trials with a maximum deviation greater than 7 cm. These trials typically involved instances where the bird's body touched a surface other than the force plate, and the trials were trimmed to analyze the section of the trial where no such instances occurred.

### Step detection and variables measured

Before segmenting data into steps, the position, velocity, force and acceleration trajectories were converted from a lab reference frame into an anatomical reference frame based on the velocity heading, such that the fore–aft direction always corresponds to the current direction of travel at the start of each stance phase. Data were then segmented into step cycles using minimum detection on the fore–aft acceleration trajectory, which we found to be the best proxy for touchdown across both walking and running speeds. This touchdown proxy was cross-checked against step counts taken from the video data. We initially used automated peak detection using the ‘findpeaks’ function in MATLAB, and then visually checked the peak detection through a user-interface that allowed iterative adjustment of the minimum peak prominence and minimum number of points between peaks until the algorithm detected a single fore-aft minimum per step.

Once the trials were segmented into step cycles, we measured the following variables at for the start and end of each step period: the position and velocity of the body COM, the position of the foot COP, and the effective length and angle of the virtual leg (calculated from the line connecting the body COM to the foot COP). From these measures, we were able to calculate initial conditions at the start of each step and the net change within each step, including the change in velocity magnitude and heading. We also measured step length, step duration, the force impulse in each direction, the minimum, maximum and mean force in each direction, the peak and mean magnitude of the resultant force vector (**F**_R_).

### Statistical analysis

All variables were normalized to dimensionless quantities before statistical analysis, based on leg length (*L*_leg_) measured as standing hip height, body mass (*M*_b_) and gravitational acceleration (***g***), according to the conventions of McMahon and Chang (Dataset 1, Table S1) (McMahon and Cheng, 1990; Daley and Birn-Jeffery, 2018). Dimensionless speed is calculated as |*V*_i_|/√(g*L_leg_), where |*V*_i_| is the magnitude of the velocity vector of the body COM at the start of each step in m s^−1^ (McMahon and Cheng, 1990; Daley and Birn-Jeffery, 2018). Normalizing to dimensionless quantities accounts for differences in variables associated with variation in body size, assuming geometric and dynamic similarity between individuals (McMahon and Cheng, 1990; Daley and Birn-Jeffery, 2018).

**Fig. 2.**
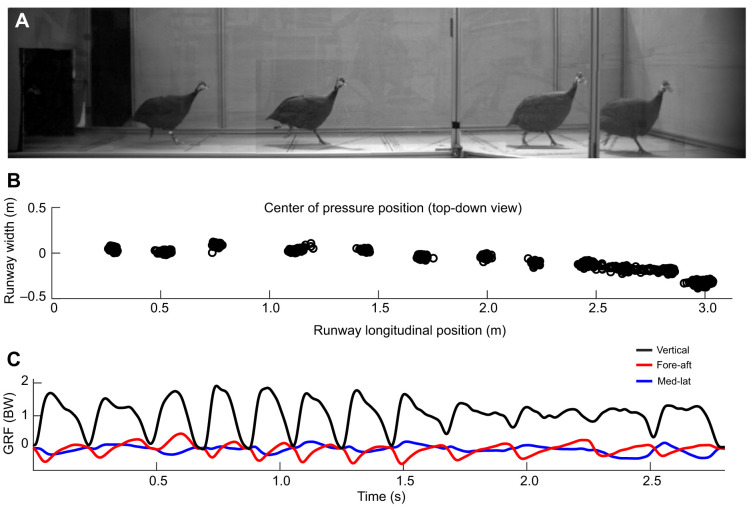
**Representative raw data for a high friction (control) turning terrain (CT) in** **the guinea fowl.** (A) A composite image from multiple frames of the video from a single trial of guinea fowl YGB moving across CT terrain. The full video is available as Movie 1. (B) The corresponding center of pressure (COP) position, shown from a top-down perspective of runway position. (C) The ground reaction forces over time throughout the trial, in the bird's anatomical frame of reference, normalized as multiples of body weight (BW) in the vertical (black), fore–aft (red) and medio–lateral (blue) directions. Vertical forces are always positive, fore–aft is negative while decelerating and positive while accelerating, and medio-lateral forces are positive pushing to the left and negative to the right.

Visual inspection of the COM trajectories revealed that birds typically initiated the turn after they reached about 1.5 m along the runway (Fig. 2). We therefore focused the statistical analysis on steps occurring after this position. We used a linear mixed-effects ANOVA with terrain condition as a main fixed factor, and individual subject as a random factor. We compared multiple models of varying complexity, and evaluated the models based on AIC and total adjusted *R*-squared. The null hypothesis model included only individual as a random effect, model 1 (null hypothesis): *Y*∼1+(1|subject). The first comparison models included condition as a fixed categorical factor, model 2: *Y*∼1+condition+(1|subject). The sharpest turning steps tended to occur near the end of the runway, so we tested a model that included a linear covariate of runway position (pos_*y*, the birds progression along the runway, measured at the start of each step) as an interaction term with terrain condition: model 3: (*Y*∼1+condition+condition*pos_*y*+(1|subject)). Finally, we also tested for learning effects with repeated trials by including trial number as a term nested within condition by individual: model 4: *Y*∼1+condition+condition*pos_*y*+(condition:trialNum|subject). Models were compared based on AIC and adjusted *R*-squared, which supported selection of model 4 delete repetition of phrase (Table S2). We calculated *F*-statistics for the effects of condition, runway position and its interaction and coefficient estimate with 95% confidence intervals (CI). We used false discovery rate (FDR) correction to calculate an adjusted *P*-value threshold to maintain a 5% false positive rate across all statistical tests (Benjamini and Hochberg, 1995). The *F*-statistics and linear mixed effects model outputs are provided in Tables S2 and S3. The mean and 95% CIs for variables in each terrain condition were calculated based on marginal means after accounting for individual random effects, reported in Table 1A. We calculated pair-wise marginal mean differences (and 95% CIs) for CT, SS and ST compared with the CS reference using the Dunnett procedure (Table 1B). The random effects term of (condition:trialNum|subject) resulted in a coefficient for the change in the variable of interest as a function of trial number within each treatment terrains, where CS is the reference terrain. We consider this coefficient to represent a learning effect of how locomotion changed with practice with repeated exposure to terrain conditions. These coefficients are reported for each individual in Table S5.

**Table 1.** Marginal means (±95% CI) for each terrain condition after removing the LME random effect of individual and pair-wise mean difference from CS reference

	Marginal means				Pair-wise mean difference		
Variable	CS	SS	CT	ST	SS	CT	ST
Speed	1.11±0.02	0.92±0.02	0.95±0.02	0.81±0.02	−0.18±0.03*	−0.15±0.03*	−0.30±0.03*
Δθ	0.3±0.6	0.2±0.6	−7.6±1.1	−6.7±0.9	−0.06±1.25	−7.90±1.32*	−6.95±1.26*
*J*_ML_	0.078±0.004	0.071±0.004	0.135±0.010	0.110±0.007	−0.007±0.011	0.057±0.011*	0.032±0.011*
**F**_ML,mean_	0.059±0.003	0.048±0.003	0.094±0.007	0.069±0.005	−0.011±0.007*	0.035±0.008*	0.010±0.007*
**F**_R,mean_	1.081±0.006	1.066±0.005	1.074±0.005	1.061±0.008	−0.015±0.010*	−0.006±0.011	−0.019±0.010*
**F**_R,max_	1.713±0.018	1.595±0.017	1.468±0.020	1.416±0.017	−0.12±0.03*	−0.24±0.03*	−0.30±0.03*
**F**_H_:**F**_V_	0.079±0.005	0.065±0.004	0.129±0.009	0.094±0.007	−0.014±0.010*	0.050±0.011*	0.016±0.010*
*T*_step_	1.34±0.01	1.54±0.02	1.47±0.03	1.70±0.03	0.20±0.04*	0.13±0.05*	0.36±0.04*
*L*_step_	1.39±0.02	1.28±0.02	1.37±0.02	1.26±0.02	−0.12±0.03*	−0.02±0.03	−0.13±0.03*
SLA_i_	104.0±0.8	109.5±0.9	94.1±0.8	89.6±1.1	5.5±1.4*	−9.9±1.5*	−14.5±1.4*
*L*_leg,mid_	1.01±0.01	1.05±0.01	1.02±0.00	1.09±0.01	0.04±0.01*	0.01±0.01	0.08±0.01*
FLA_mid_	89.4±0.4	89.6±0.3	86.3±0.4	85.4±0.7	0.2±0.6	−3.1±0.7*	−4.0±0.6*
*N*	367	391	330	385			

Asterisks indicate statistically significant differences from CS.

## RESULTS

### Overview of turning strategies

**Fig. 3.**
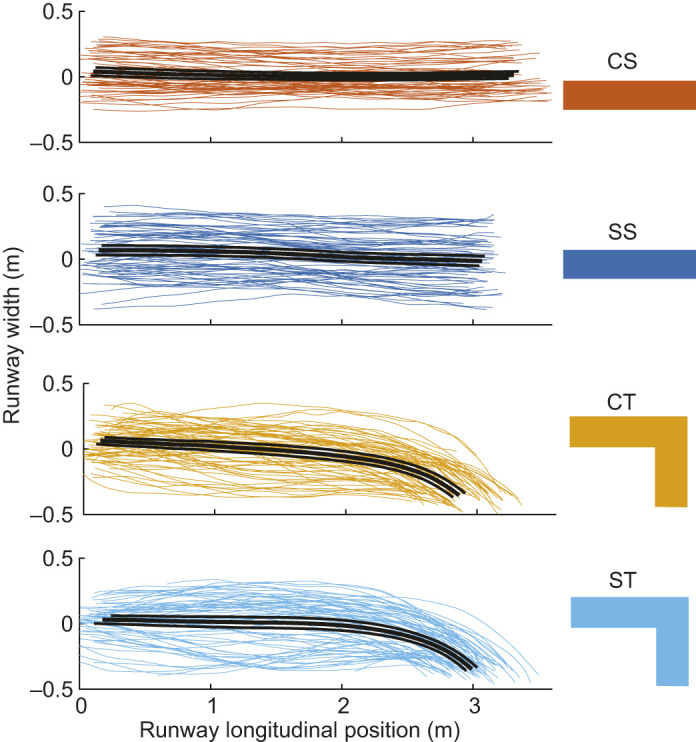
**Top-down** **view of body center of mass (COM) position trajectories across the four terrain conditions.** Control straight (CS, top, orange), slippery straight (SS, dark blue), control turns (CT, yellow) and slippery turns (ST, light blue). Most of the turn maneuvers occurred after the birds reached 1.5–2 m along the runway.

Guinea fowl executed straight runs and turns, with the turning runway wide enough to allow selection of a range of paths and turn sharpness. GRF data from a representative trial illustrate a typical turn maneuver (Fig. 2 and see Movies 1,2). Overhead trajectories of the body COM position suggested that birds use similar paths between control turn (CT) and slippery turn (ST) terrains (Fig. 3). The average change in velocity heading (Δθ) per step was −7.6±1.1 deg in CT and −6.7±0.9 deg ST (mean±95% CI, Table 1), differing significantly from CS, but not between CT and ST (Table 1). This does not support the prediction of more gradual turns in ST. The linear mixed effects (LME) model indicated a significant effect of terrain (*F*=21.0, *P*<0.001) and the interaction Terrain×Runway position (*F*=45.9, *P*<0.001) on Δθ. The interaction term revealed that the birds turned significantly more sharply as they approached the end of the runway in both CT and ST compared with CS and SS (*P*<0.001 for both CS and ST compared with CS; *P*=0.84 for SS compared with CS). The interaction coefficients (Tables S2,S3) suggested slightly more gradual turns near the end of the runway in ST compared with CT; however, the difference was not statistically significant.

### Speed modulation

**Fig. 4.**
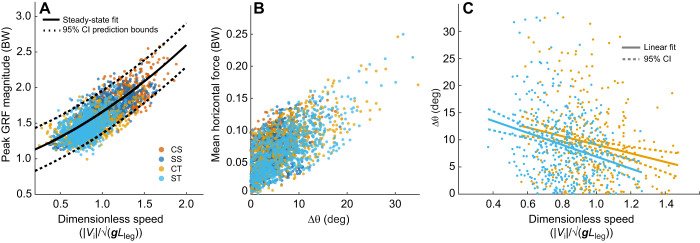
**Force magnitudes, speed and turn sharpness in turning terrain compared with straight steps.** (A) Running speed (|*V*_i_|) against **F**_max_, with both quantities normalized to dimensionless quantities. A second order polynomial fit with 95% CI for prediction bounds is shown for control straight (CS). In turning and slippery terrains, most steps remain within the prediction bounds of the CS speed–force relationship. (B) Mean |**F**_H_| against change in velocity heading per step (Δθ). Mean |**F**_H_| increases with Δθ consistent with the point-mass model (Fig. 1C), but most steps involve a small Δθ. (C) Running speed against Δθ in the control turn (CT) and slippery turn (ST) terrains. The solid line indicates the linear fit for each terrain (±95% CI for the fit indicated by dashed line).

Guinea fowl ran significantly slower in turns and slippery terrain. The LME indicated a significant effect of terrain on speed (*F*=8.6, *P*<0.001, Tables S2,S3). Mean speed was 1.11±0.02 (mean±95% CI) in CS, 0.95±0.02 in CT and 0.81±0.02 in ST, corresponding to 14% and 27% slower speed relative in CT and ST relative to CS (Table 1A). Slippery straight (SS) speed was 0.92±0.02, 17% slower than CS, intermediate between CT and ST. Slower speeds in slippery terrains are consistent with the slip avoidance hypothesis (H2). There was a significant negative relationship between running speed and Δθ, indicating that sharper turn steps occur at slower speeds (Fig. 4C). The slope was −7.9±4.02 for CT and −11.0±4.06 for ST (95% C.I), slightly more negative for ST compared with CT but the difference was not statistically significant.

### Force modulation

**Fig. 5.**
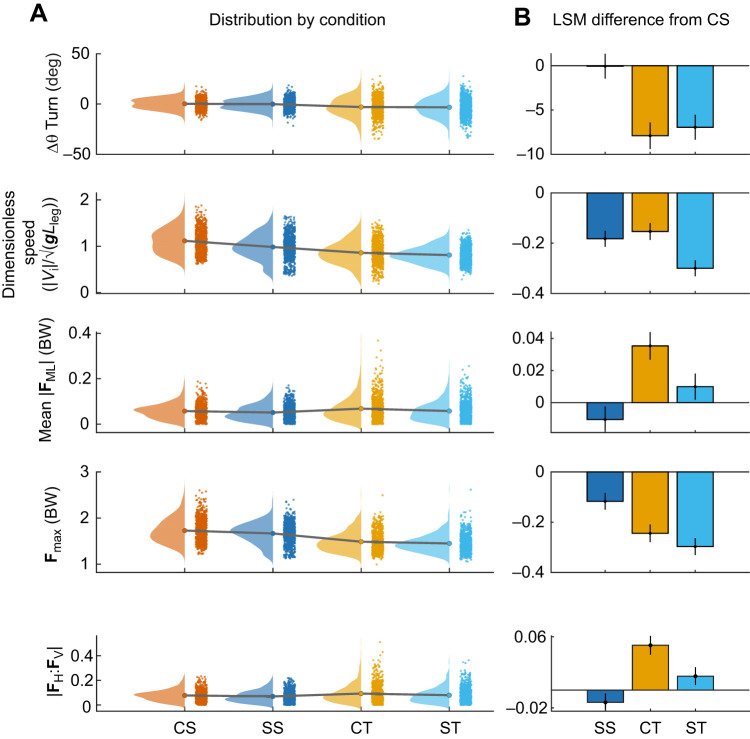
**Measurement of turning dynamics across conditions.** (A) Distributions for the key measures of turning dynamics in dimensionless quantities: Δθ (deg), dimensionless speed (|*V*_i_|/√(g*L_leg_)), mean |**F**_ML_| in body weight (BW), **F**_max_ (in BW) and the mean ratio of horizontal to vertical force (|**F**_H_:**F**_V_). Terrain abbreviations and colors as in previous figures. (B) Least-squared mean differences (±95% CI) for the pair-wise comparisons of SS, CT and ST with the control straight (CS) condition.

A consistent relationship was maintained between peak ground reaction force magnitude (**F**_R,max_) and speed across terrains, consistent with the force regulation hypothesis (H1) (Fig. 4A). **F**_R,max_ averaged 1.71±0.02 BW in CS terrain. **F**_R,max_ was −0.24±0.03 BW lower in CT (−14%) and −0.30±0.03 lower in ST (−17%) (Table 1, Fig. 5), while remaining within the 95% prediction interval of the speed–force relationship of CS trials (Fig. 4A). **F**_R,max_ was −0.12±0.03 BW lower in SS compared with CS (Table 1B, Fig. 5).

As expected, turning involved a higher **F**_ML_ with significant effect of Terrain and Terrain×Runway position on ML impulse (Terrain: *F*=6.1, *P*=0.0004; Interaction: *F*=17.8, *P*<0.001, Table S3) and mean force (**F**_ML,mean_, Fig. 4B, Terrain: *F*=6.4, *P*=0.0003, Interaction *F*=16.8, *P*<0.001, Table S3, Fig. 5). Given the gradual turn angles observed, small increases in **F**_ML,mean_ are expected based on the impulse-momentum equations (Eqns 1–4), which show that force demands scale with sin(Δθ). Consistent with this, increases in **F**_ML,mean_ were small in magnitude: +0.035±0.008 BW higher in CT and +0.010±0.007 BW higher in ST relative to CS (Table 1B, Fig. 5). In SS, **F**_ML,mean_ was −0.011±0.007 BW lower than CS (Fig. 5, Table 1B). The lower values of **F**_ML,mean_ in ST compared with CT, and SS compared with CS suggest adjustments to gait in slippery terrain to reduce ML forces for slip avoidance (H2).

The mean ratio of horizontal-to-vertical force (|**F**_H_|:**F**_V_) increased in turns compared with straight runs, as expected, but was significantly lower in ST compared with CT, consistent with the slip avoidance hypothesis (H2). The LME indicates a significant effect of Terrain (*F*=6.9, *P*=0.0001) and Terrain×Runway position (*F*=17.8, *P*<0.001) on |**F**_H_|:**F**_V_. As expected, the ratio increased in turns compared with straight runs: the ratio was 0.050±0.011 higher in CT (+64%) and 0.016±0.010 higher in ST (+20%) compared with CS (Table 1, Fig. 5). In slippery straight runs (SS), |**F**_H_|:**F**_V_ was −0.014±0.010 lower (−17%) compared with CS (Table 1, Fig. 5). Lower |**F**_H_|:**F**_V_ in slippery terrains compared with control terrains suggests adjustments for slip avoidance (H2).

#### Changes in gait and posture

**Fig. 6.**
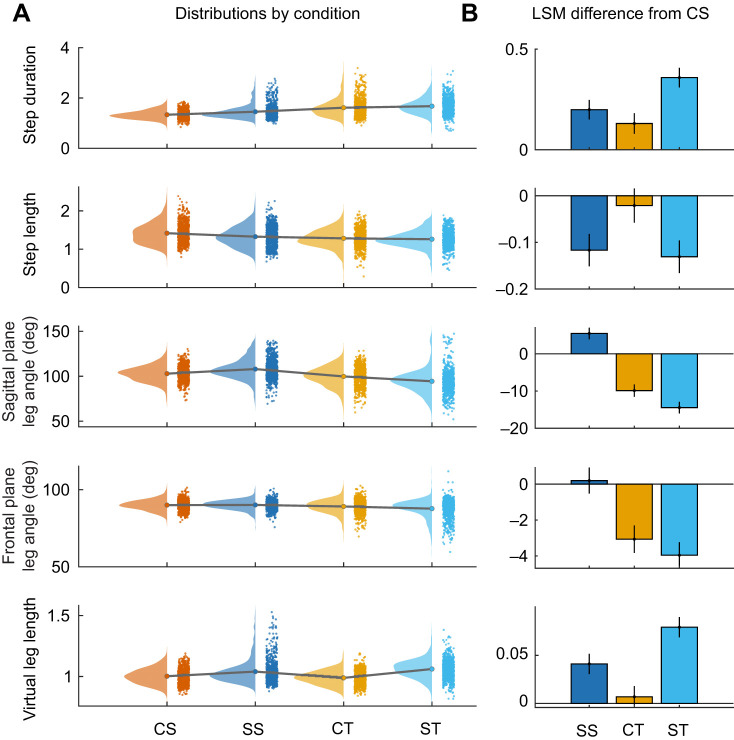
**Measurement of gait and posture across conditions.** (A) Distribution of gait and posture measures in dimensionless quantities: step duration, step length, sagittal plane leg angle, frontal plane leg angle, and virtual leg length. Terrain condition abbreviations and colors as in previous figures. (B) Least-squared mean differences (±95% CI) for the pair-wise comparisons of SS, CT and ST with the control straight (CS) condition.

Frontal plane leg angle decreased in turning terrains, by −3.1±0.7 in CT and −4.0±0.6 in ST compared with CS (Fig. 6, Table 1B) indicating that the virtual leg was angled toward the inside of the turn (i.e. the bird leaned into the turn). The LME revealed significant effects for Terrain (*F*=118.8, *P*<0.001) and the interaction Terrain×Runway position (*F*=178.9, *P*<0.001), indicating that the birds leaned more as they approached the end of the runway (Table S4). Step duration increased in turn and slippery terrains compared with control straight conditions (Fig. 6). The LME indicates a significant effect of Terrain on step duration (*F*=5.1, *P*=0.0016), but not Runway position or their interaction (Table S3). Mean step duration (in dimensionless quantities) was 1.34±0.01 in CS, and increased by +0.13±0.05 in CT, +0.36±0.04 in ST and +0.20±0.04 in SS, increases of 10%, 27% and 15%, respectively (Table 1B, Fig. 6), with larger shifts in ST and SS relative to CT. Increasing step duration allows for horizontal forces to be distributed over longer durations, which may help limit |**F**_H_|:**F**_v_ for slip avoidance (H2). Changes in step length and leg posture included significant effects of Terrain, Runway position and the Interaction terms (Table S2). Step length was not significantly different between CS and CT (Table 1) but decreased by −0.13±0.03 *L*_leg_ in ST and −0.12±0.03 *L*_leg_ in SS reflecting decreases of 9 and 8%, respectively (Table 1B). This suggests reducing step length for slip avoidance (H2). Midstance virtual leg length increased in slippery terrains relative to control, by +0.08±0.01 *L*_leg_ in ST and +0.04±0.01 in SS (Table 1B). This suggests adopting a more upright posture with shorter steps for slip avoidance (H2).

#### Changes in ground reaction force profile

**Fig. 7.**
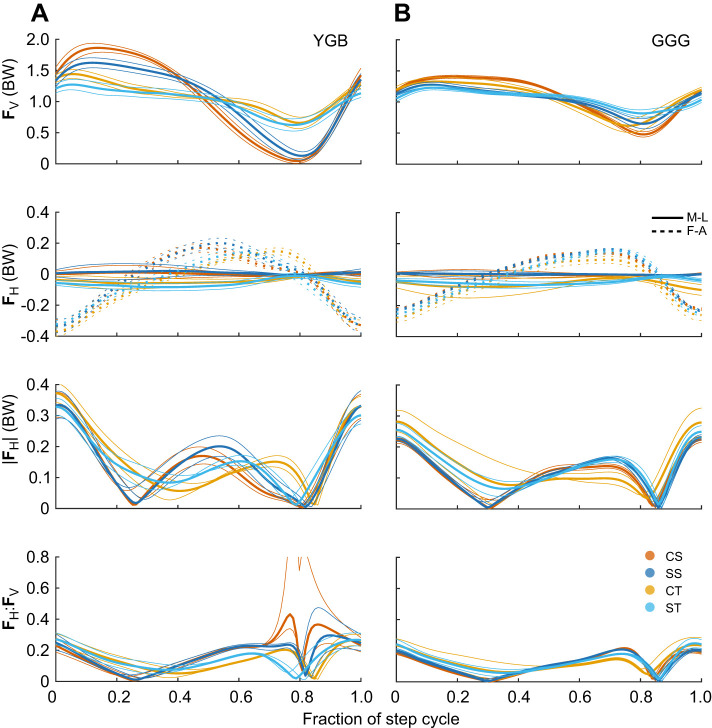
**Average ground reaction force trajectories (±95% CI) as a fraction of the step cycle for two individual guineau fowl.** Results for YGB (left) and GGG (right), across terrain conditions. Vertical forces (top) are always positive, **F**_FA_ (second row, dashed) is negative for decelerating and positive for accelerating. **F**_ML_ (second row, solid) is positive pushing to the left and negative to the right. The vector magnitude of horizontal forces (|**F**_H_|, third row) and the ratio of |**F**_H_|:**F**_V_ (bottom row) is relevant to fall risk (Eqns 5–6). Individual YGB had the highest average speed and GGG had the slowest. Accordingly, YGB exhibits higher peaks and lower minima in vertical force compared with GGG.

Changes in gait were associated with differences in the GRF trajectory among conditions (Fig. 7). In CS terrain, speed was sometimes fast enough to include an aerial phase, with **F**_v_ showing a distinct force peak and falling to zero between contacts; however, this varied by individual. In turning steps, **F**_v_ had a longer plateau compared with CS and vertical force remained above 0.4–0.5 BW throughout the step cycle, indicating no aerial phase. Fluctuations in **F**_v_ are lower at slower speeds and longer step durations, with lower peaks and higher minimum. The ratio |**F**_H_|:**F**_v_ is typically highest near the end of stance when **F**_v_ rapidly declines to a minimum, whereas |**F**_H_| remains high for longer. Maintaining higher minimum **F**_v_ allows birds to maintain a lower ratio |**F**_H_|:**F**_v_ in late stance in CT and ST, despite the increase in |**F**_ML_| and |**F**_H_| (Fig. 7). This enables higher frictional forces while executing turns.

#### Individual variation in speed and ground reaction forces

**Fig. 8.**
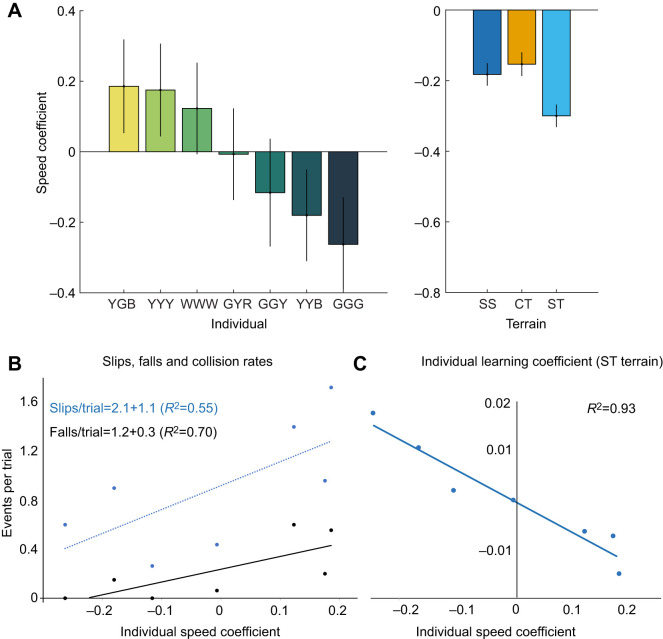
**Individual variation in speed and error rates in seven guineau fowl.** (A) Individual speed coefficients compared with the terrain coefficients from the LME model. (B) There is a positive correlation between individual speed coefficient and number of slips (blue) and fall or collision events (black). Faster individuals experienced more errors. (C) Individual learning coefficients correlated negatively with individual speed coefficient. Individuals who ran faster slowed down with practice, whereas individuals who ran slower sped up with practice.

Some individuals moved faster or slower than the others on average across all conditions, indicated by the individual random effect coefficients for speed (Fig. 8A). The magnitude of the random effect coefficients was comparable to the terrain condition effects (Fig. 8A). Associated with these differences in preferred speed, there was substantial variation in GRF trajectories as illustrated by comparing the slowest and fastest individuals in Fig. 7. Faster individuals had higher peak and lower minima in **F**_v_, and higher variation in the |**F**_H_|:**F**_v_. Slower individuals had smaller **F**_V_ fluctuations and lower variation in |**F**_H_|:**F**_v_ (Fig. 7).

#### Occurrence of slips and falls

In the CT terrain, five slips, one fall and one collision event occurred across 130 trails. In the ST terrain, 111 slips, 23 falls and two collisions occurred across 128 trials. However, these events were not evenly distributed among individuals. Two of seven individuals had a fall or collision in 60% of their trials, whereas two others never experienced a fall or collision (Fig. 8). There was a significant correlation between the individual speed coefficient and the number of slips or falls in the ST terrain (Fig. 8B). We also observed a significant negative correlation between the individual speed coefficient and the learning effect coefficient, the coefficient for changes in speed with practice across repeated trials (Fig. 8D). Individuals that ran faster on average slowed down with practice and those that ran slower sped up with practice. However, the magnitude of the learning coefficient was small and individual variation in preferred speed persisted across terrains and trials.

## DISCUSSION

We investigated how guinea fowl adjust locomotor strategies to manage the trade-off between speed and maneuverability while navigating turns in high and low friction terrains. The runways were wide enough to allow the bird to select a range of paths and turn sharpness to compare preferred strategies between conditions. Consistent with the force regulation hypothesis (H1), we found a consistent relationship between peak GRF and speed across terrains, and slower speed in turns compared with straight runs. Based on the slip avoidance hypothesis (H2), we predicted that guinea fowl would use slower speed and shallower turn angles in slippery turns. Guinea fowl did use slower speed in slippery terrains, with slippery turns slower than control turns, and slippery straight slower than control straight terrain (Table 1), suggesting shifts in preferred speed for slip avoidance. Surprisingly, guinea fowl used similar turn sharpness in high and low friction terrains, with gradual turns of Δθ of ∼7 deg per step (Table 1). Guinea fowl exhibited changes in posture in turns, with a more vertical leg orientation in the sagittal plane and a decrease in frontal plane leg angle, indicating that they leaned into the turn to avoid toppling. In slippery terrain, guinea fowl used shorter steps and longer virtual leg length, suggesting a more upright posture with shorter steps for slip avoidance. This is consistent with the finding by Clark and Higham (2011) that guinea fowl avoid falls on slippery surfaces when they run with a more upright, vertically oriented posture. Overall, the slow speeds and gradual turn sharpness suggests that guinea fowl used a relatively cautious approach that did not push maneuverability limits in the conditions measured here.

The tight coupling between speed, turn sharpness and ground reaction forces across conditions suggests that speed modulation serves as an effective mechanism for managing slip and fall risks in maneuvers. Vertical force fluctuations are tightly coupled to speed, with smaller fluctuations at slower speeds, with peak and the minimum values closer to body weight (Birn-Jeffery and Daley 2018). The ratio of horizontal to vertical force (|**F**_H_|:**F**_V_) reaches peak values near the end of stance, when vertical forces rapidly decline (Fig. 7; Daley and Birn-Jeffery, 2018). By moving at slower speeds, vertical force can be maintained at higher levels in late stance, approaching the step-to-step transition when horizontal forces are high, and this may allow horizontal forces to be applied for longer while maintaining |**F**_H_|:**F**_V_ below friction limits, to avoid slipping. More broadly, speed control appears to be a fundamental mechanism by which animals manage mechanical demands and risks while navigating their environment (Wheatley et al., 2021). Moving fast enables animals to better compete for resources in their environment and avoid predation, but can risk accidents such as collisions, falls and injury; whereas moving slowly reduces some risks and allows time to evaluate how to navigate the environment (Clemente and Wilson, 2015; Wheatley et al., 2015; Wynn et al., 2015; Amir Abdul Nasir et al., 2017). In the current study, the primary motivation for movement was reaching the safety box at the end of the runway, where the guinea fowl had access to food, water and a nearby companion. This is not incentive enough to motivate fast speeds in the same manner as predator escape in the wild (Wilson et al., 2018). Additionally, the specific terrain conditions presented here differ from the complex and variable substrates animals encounter in the wild. Nonetheless, our findings on submaximal turning in controlled conditions provides insight into how animals adjust movement to balance demands for speed, maneuverability and safety.

In both the high and low friction terrain conditions measured here, guinea fowl executed turns gradually over a distance corresponding to ∼5–6 steps, rather than executing a sharp cutting maneuver. This contrasts with shorter timescales of observed in locomotion over uneven terrains, which involve adjustments only 1–2 steps ahead of a vertical height perturbation (Birn-Jeffery and Daley, 2012; Matthis and Fajen, 2013; Birn-Jeffery et al., 2014; Gordon et al., 2015; Matthis et al., 2017; Matthis et al., 2018). The difference between turning and obstacle negotiation suggests that timescales of adjustments might depend on the spatial scale of the terrain features (Clifton et al., 2023). While discrete obstacles may require only short-term adjustments, directional changes to avoid wall collisions might require coordinated changes over longer time scales. However, the specific sensorimotor control mechanisms underlying path planning in guinea fowl remain unknown and would be an interesting topic for future investigation. Another possible factor in the sharpness of turning may be the energetic cost of maneuvers. Recent work in humans suggests that turning paths can be predicted based on optimization to minimize energetic cost of travel, and it would be interesting in future work to test whether turning paths in guinea fowl are likewise consistent with minimizing cost (Brown et al., 2021).

The persistent individual variation in preferred speed and the rate of slips and falls suggests that movement strategies may be influenced by variation in behavioral tendencies including risk perception and risk tolerance. All individuals experienced slipping in at least 30% of trials in slippery turns, and some slipped as many as 1.7 times per trial on average (Fig. 8). Individuals that ran faster experienced higher incidence of slips and falls (Fig. 8). Two of seven individuals had a fall or collision in 60% of trials, but two other individuals experienced no falls or collisions. Individuals that ran faster did slow down slightly with practice, and individuals that ran slowly sped up with practice. Nonetheless, the learning effects were small compared with the individual differences in preferred speed, which persisted with repeated exposure to slippery terrains. This finding suggests that locomotor strategies in novel conditions may be influenced by behavioral tendencies including risk perception and tolerance. However, the specific mechanisms underlying behavioral variation in guinea fowl are multi-factorial and largely unstudied and would be an interesting area for future investigation. Several factors might cause individuals to respond differently to environmental risks, including muscle strength and power capacity, energy reserves, prior experience, bold/shy personality expression and explore/exploit tendencies (Dall et al., 2004; Adriaenssens and Johnsson, 2011; Dingemanse and Dochtermann, 2013; MacKay and Haskell, 2015; Seebacher et al., 2015). These factors could influence the response to risk and the relative weighting of priorities, including minimizing energy demands, minimizing injury risk or minimizing time out in the open, visible to predators. In future work, it would be interesting to explore model-based predictions of maneuvering that accounts for individual variation in risk tolerance and reward seeking (Hackett et al., 2020; 2022; Brown et al., 2021; Seethapathi et al., 2024). Models used in combination with experimental data could test how individual differences in movement patterns relate to physical, physiological and cognitive traits and how strategies shift under different motivational states, such as predator escape versus foraging (Wheatley et al., 2018; 2020).

The relatively gradual turning strategies observed in our guinea fowl suggest that bipedal animals may face greater constraints on turning maneuverability compared with quadrupeds and other multi-legged animals. Our findings are consistent with reduced speeds in humans on curved paths (McMahon and Greene, 1979; Greene, 1985; Usherwood and Wilson, 2006; Chang and Kram, 2007; Churchill et al., 2016; Taboga et al., 2016). Previous work on quadrupeds, hexapods and other many-legged animals suggests that they may have a greater ability to simultaneously maintain speed and stability in turns compared with bipeds, because they have flexibility to redistribute forces among legs (Jindrich and Full, 1999; Walter, 2003; Usherwood and Wilson, 2005; Jindrich et al., 2007; Daley, 2016). Foot morphology and foot pad properties might also contribute to variation among species in ability to exert frictional forces for turning maneuvers; however, examining this requires detailed measurements of foot–substrate interactions (Zhang et al., 2021). Drawing general conclusions is challenging because maneuvering performance has not been directly compared in the same conditions across species. Ostriches were found to take sharper turns than measured for guinea fowl, with a Δθ of 18 deg per step (Jindrich et al., 2007). However, the conditions were not the same between studies – ostriches were presented with a sudden, unexpected barrier to forward progression, forcing a sharp turn; whereas the guinea fowl were presented with a wide, visible L-shaped runway that allowed selection of turn sharpness. In future work, it would be interesting to compare maneuvering performance and foot–substrate interactions in comparable conditions across diverse species to investigate how body size, leg number and foot morphology influence performance trade-offs between speed, maneuverability, stability and safety in maneuvering.

### Conclusions

Shifts in locomotor strategies between high and low friction turning terrains suggest force regulation via speed control as a general strategy for balancing trade-offs among mechanical demands in unsteady maneuvers. By slowing down, fluctuations in vertical force are reduced, which helps to avoid large peaks in the ratio of horizontal to vertical forces in late stance. This reduces the risk of exceeding the available frictional forces while turning. We observed that substantial variation in preferred locomotor speeds among individuals persisted across conditions with practice and these differences correlate with individual variation in slip and fall rates. These findings suggest that it is important for future studies to investigate how individual variation in risk perception, motivation and capacity may influence locomotor priorities and movement strategies in unsteady tasks.

## Supplementary Material

10.1242/jexbio.250929_sup1 Supplementary information

Dataset 1. Spreadsheet of data derived from experimental data that was used for the statistical analysis. Note that values are normalized to dimensionless quantities, as described in the manuscript text. The columns included are specified below.
