# Supplementary material for: Faster fowl fall frequently: speed and force regulation during turning maneuvers by guinea fowl on high and low friction terrains
Source: J Exp Biol. 2026 Mar 30;229(6):jeb250929. doi: 10.1242/jeb.250929 (PMC13086494; doi:10.1242/jeb.250929)
Supplement: Supplementary information [file jexbio-229-250929-s1.pdf]

Table S1. Linear mixed effects model selection

Model		adj. R^2	AIC
1	V_i ~ 1+ (1|subject)	0.27	-268.8
2	V_i ~ 1+ condition + (1|subject)	0.42	-601.0
3	V_i ~ 1+ condition + condition*pos_y + (1|subject)	0.45	-673.1
4	V_i ~ 1+ condition + condition*pos_y + (condition:trialNum|subject)	0.59	-1033.6

Table S2. Linear mixed effects model results, based on model 4 (see methods in main text). Degrees of freedom condition= 3, i_pos_y = 1, conditions:i_pos_y = 3, error = 1465. Asterisks indicate statistical significance after FDR correction, which resulted in a threshold value of $p < 0.0183$.

	F-statistics		
Variable	Terrain	y-Pos	Interaction
speed	**8.6***	**32.5***	0.3
Δθ	**21.0***	0.1	**45.9***
Impulse_ML	**6.1***	0.0	**17.8***
Fmean_ML	**6.4***	0.0	**16.8***
Fmean_R	0.8	2.1	0.8
Fmax_R	**7.0***	**7.2***	2.4
F_H:F_V	**6.9***	0.0	**17.8***
stepDur	**5.1***	1.2	1.8
stepLength	**3.9***	**48.8***	**3.6***
i_SLA	**48.2***	**21.0***	**134.5***
m_LL	**27.6***	**122.7***	**34.9***
m_FLA	**118.8***	**5.6***	**178.9***

Journal of Experimental Biology • Supplementary information

Table S3. Linear mixed effects model coefficients (± 95% confidence intervals) from model 4 (see methods).

Linear mixed effects model coefficients

Variable	(Intercept)	SS	CT	ST	i_pos_y	SS:i_pos_y	CT:i_pos_y	ST:i_pos_y
speed	1.33±0.10	-0.20±0.14	-0.23±0.13	-0.31±0.13	-0.10±0.03	0.02±0.06	0.02±0.05	0.00±0.05
Δθ	0.68±3.64	19.21±5.80	-0.60±5.34	12.28±5.31	-0.18±1.51	-12.17±2.55	0.24±2.30	-8.63±2.29
Impulse_ML	0.076±0.031	- 0.093±0.050	- 0.002±0.046	- 0.059±0.046	0.001±0.013	0.067±0.022	- 0.002±0.020	0.041±0.020
Fmean_ML	0.057±0.021	- 0.067±0.034	- 0.008±0.031	- 0.046±0.032	0.001±0.009	0.046±0.015	- 0.001±0.013	0.025±0.013
Fmean_R	1.10±0.04	-0.02±0.05	-0.03±0.05	0.00±0.05	-0.01±0.01	0.01±0.02	0.01±0.02	-0.01±0.02
Fmax_R	1.82±0.10	-0.15±0.14	-0.22±0.13	-0.29±0.13	-0.05±0.04	-0.04±0.06	0.05±0.06	0.00±0.06
F_H:F_V	0.077±0.030	- 0.092±0.048	- 0.010±0.043	- 0.070±0.043	0.001±0.012	0.064±0.021	- 0.002±0.018	0.038±0.018
stepDur	1.28±0.15	-0.07±0.21	0.23±0.19	0.28±0.19	0.03±0.05	0.09±0.09	-0.01±0.08	0.03±0.08
stepLength	1.69±0.10	-0.23±0.15	-0.18±0.14	-0.12±0.14	-0.13±0.04	0.10±0.06	0.03±0.06	-0.01±0.06
i_SLA	95.2±4.3	22.4±6.7	-3.8±6.3	30.0±6.3	4.0±1.7	-14.5±2.9	4.2±2.6	-20.0±2.6
m_LL	0.87±0.03	0.03±0.05	-0.13±0.04	0.07±0.04	0.06±0.01	-0.01±0.02	0.08±0.02	0.01±0.02
m_FLA	87.3±1.9	9.8±3.1	1.0±2.8	24.3±2.8	0.9±0.8	-5.8±1.3	-0.4±1.2	-12.7±1.2

Table S4. Coefficients for random effects term of condition:trialNum|subject (trial number within condition by subject) on running speed (vector magnitude of center of mass velocity), with control straight (CS) trials as the reference condition, and coefficients calculated for slippery straight (SS), control turns (CT), and slippery turns (ST) as a function of trial number.

Individual	SS	CT	ST
GGG	-0.0010378	0.01148819	0.0176442
GGY	0.02269599	-0.0351337	0.00187434
GYR	-0.0071413	0.00876865	-7.22E-06
WWW	0.00365244	-0.0037298	-0.0064067
YGB	-0.0043608	-0.0176093	-0.0151119
YYB	0.01243366	-0.0001475	0.01061256
YYY	0.0071079	-0.002161	-0.0073752

Table S5. Morphological variables and normalization quantities used to convert data to dimensionless quantities, following conventions of according to the conventions of McMahon and Chang (McMahon & Cheng 1990; Daley & Birn-Jeffery 2018). Dimensionless quantities were calculated by dividing each variable by the subject-specific values of the quantity with the same time of unit in the table below (length, force, speed, time, impulse).

**indIDs**	**Leg norm (hip height, m)**	**Body mass (kg)**	**Force norm (N)**	**Speed, norm (m/s)**	**Time norm (s)**	**Impulse norm (Ns)**
GGG	0.189	2.16	21.19	1.36	0.14	2.94
GGY	0.188	1.98	19.42	1.36	0.14	2.69
GYR	0.198	1.71	16.78	1.39	0.14	2.38
WWW	0.187	1.92	18.84	1.35	0.14	2.60
YGB	0.195	2.09	20.50	1.38	0.14	2.89
YYB	0.197	1.57	15.40	1.39	0.14	2.18
YYY	0.199	1.8	17.66	1.40	0.14	2.51

Journal of Experimental Biology • Supplementary information

Dataset 1. Spreadsheet of data derived from experimental data that was used for the statistical analysis. Note that values are normalized to dimensionless quantities, as described in the manuscript text. The columns included are specified below.

date	Date of recording
subject	Guinea fowl subject ID
condition	Runway condition (Control Turn (CT), Slippery Turn (ST), Control Straight (CS) and Slippery Straight (SS)
trialNum	Trial number based on the total number of trials experienced by that individual in the specific terrain.
stepNum	Step number within the trial
stepDur	Step duration, measured from foot contact to the next foot contact.
i_pos_x	Initial Center of Mass (COM) position for each step, in the medio-lateral direction relative to the current heading.
i_pos_y	Initial COM position for each step, which indicates the total distance traveled along the runway
i_pos_z	Initial COM position for each step, in the vertical direction relative to the current heading
d_pos_x	Change in medio-lateral CoM position during the step
d_pos_y	Change in fore-aft COM position during the step
d_pos_z	Change in vertical COM position during the step
i_vel_x	Initial medio-lateral COM velocity for the specific step
i_vel_y	Initial fore-aft COM velocity for the specific step
i_vel_z	Initial vertical COM velocity for the specific step
d_vel_x	Change in medio-lateral COM velocity during the specific step
d_vel_y	Change in fore-aft COM velocity during the specific step
d_vel_z	Change in vertical COM velocity during the specific step
i_heading	Initial CoM velocity heading for the specific step, in degrees
d_heading (Δθ)	Change in CoM velocity heading during the specific step, in degrees. Negative values indicate turning toward the right relative to the birds initial heading.
impulse_x	Force impulse (integration of force over time), in the x direction
impulse_y	Force impulse, in the y direction
impulse_z	Force impulse in the z direction
Fmax_x	Peak force in the x direction within the step period
Fmax_y	Peak force in the y direction within the step period
Fmax_z	Peak force in the z direction within the step period
Fmax_R	Peak magnitude the resultant ground reaction force vector within the step period
Fmin_x	Minimum force in the x direction within the step period
Fmin_y	Minimum force in the y direction within the step period
Fmin_z	Minimum force in the z direction within the step period
Fmin_R	Minimum of the resultant ground reaction force vector within the step period
stepLength	Distance travelled during the step
speed (velMag)	Magnitude of the velocity vector at the start of the step
i_LL	Initial virtual leg length at the start of the current step, measured from the Center of Pressure (CoP) to the CoM
i_SLA	Initial sagittal plane leg angle at the start of the current step, measured based on the angle of the virtual leg between the CoP and the CoM
i_FLA	Initial frontal plane leg angle at the start of the current step, measured based on the angle of the virtual leg between the CoP and the CoM
m_LL	Mid-stance virtual leg length for the current step, measured from the Center of Pressure (CoP) to the CoM
m_SLA	Mid-stance sagittal plane leg angle for the current step measured based on the angle of the virtual leg between the CoP and the CoM
m_FLA	Mid-stance frontal plane leg angle for the current step measured based on the angle of the virtual leg between the CoP and the CoM
F_H:F_V	Ratio of horizontal force impulse magnitude to vertical force impulse during the step. Since these are measured over the same time period, it is equivalent to the ratio of mean horizontal and mean vertical force.
Fmean_H	Average horizontal force magnitude (vector magnitude of medio-lateral and fore-aft forces) over the current step
Fmean_ML	Average magnitude of medio-lateral force over the current step
Fmean_V	Average vertical force over the current step
Fmean_R	Average resultant force magnitude (vector magnitude of the medio-lateral, fore-aft and vertical forces) over the current step.

Available for download at
https://journals.biologists.com/jeb/article-lookup/doi/10.1242/jeb.250929#supplementary-data

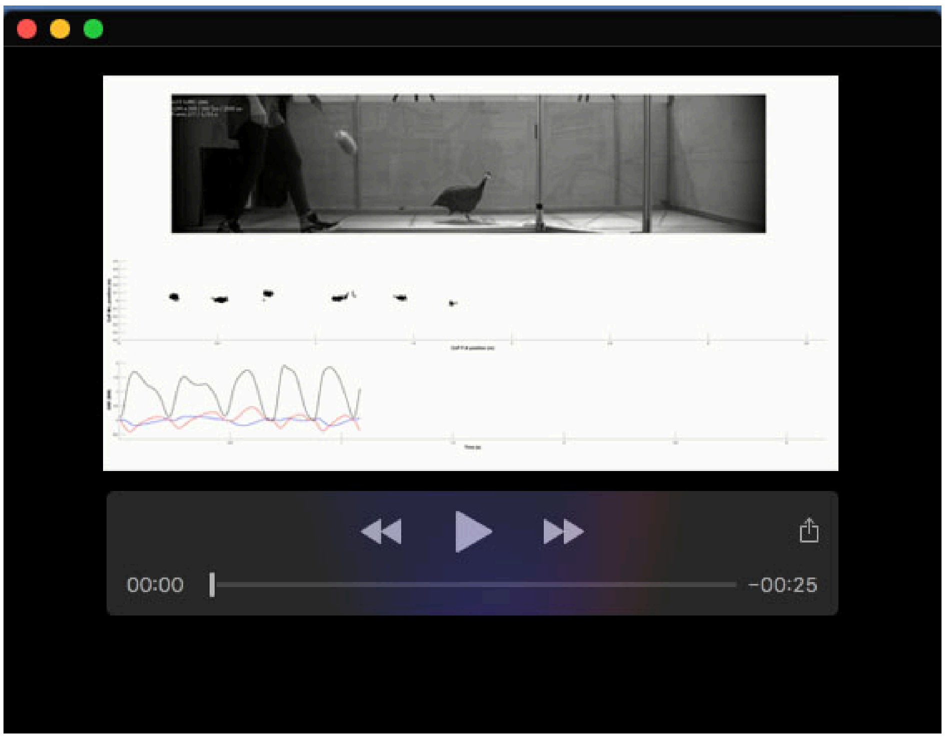

Movie 1. Guinea fowl YGB navigating the control (high friction) 90-degree turn runway.

A representative movie (top panel) synchronized with the corresponding center of pressure position data (middle), shown from a top-down perspective of runway position (longitudinal progression on the x-axis and runway width on the y-axis), and ground reaction forces (bottom) over time throughout the trial, normalized as multiples of body weight (BW) in the vertical (black), fore-aft (red) and medio-lateral (blue) directions. Vertical forces are always positive, fore-aft is negative while decelerating and positive while accelerating, and medio-lateral forces are positive pushing to the left and negative to the right.

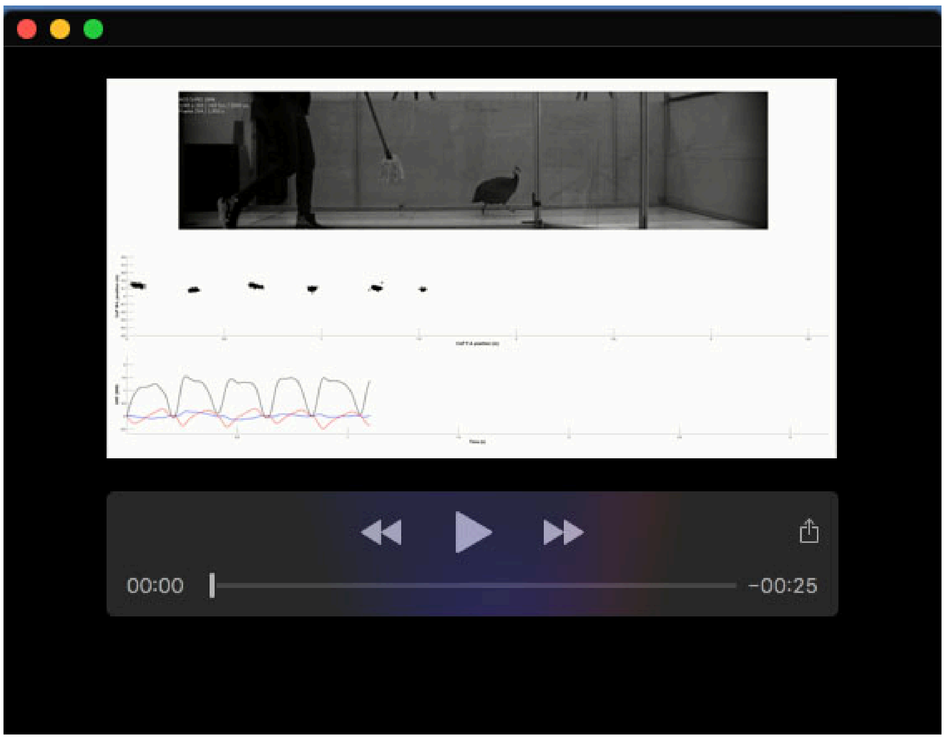

Movie 2. Guinea fowl YGB navigating the slippery (low friction) 90-degree turn runway.

A representative movie (top panel) synchronized with the corresponding center of pressure position data (middle), shown from a top-down perspective of runway position (longitudinal progression on the x-axis and runway width on the y-axis), and the ground reaction forces (bottom) over time throughout the trial, normalized as multiples of body weight (BW) in the vertical (black), fore-aft (red) and medio-lateral (blue) directions. Vertical forces are always positive, fore-aft is negative while decelerating and positive while accelerating, and medio-lateral forces are positive pushing to the left and negative to the right. Note that in this specific trial, the bird slips and falls (body touches the ground) in the corner of the turn.
